# Traumatic Brain Injury-Associated Biomarkers for Pediatric Patients

**DOI:** 10.3390/children12050598

**Published:** 2025-05-04

**Authors:** Solonas Symeou, Spyridon Voulgaris, George A. Alexiou

**Affiliations:** Department of Neurosurgery, School of Medicine, University of Ioannina, 45500 Ioannina, Greece; md07011@uoi.gr (S.S.); svoulgar@uoi.gr (S.V.)

Traumatic brain injury (TBI) is a major public health concern and remains one of the leading causes of both acute and long-term morbidity, as well as mortality in children worldwide [1]. Recent studies revealed that in the US, pediatric TBI (pTBI) accounts for over 500.000 emergency department (ED) visits and approximately 7.000 deaths annually [2]. Similarly, in Europe, the burden remains substantial, with 441.368 hospital discharges, which are higher among children aged 0–4, and 2.303 recorded deaths, with the highest mortality observed in adolescents aged 15–19 [3].

Pediatric TBI spans a spectrum of severity, ranging from mild concussions to devastating diffuse axonal injuries. However, unlike adults, children exhibit distinct injury patterns and mechanisms, and one should remember also the presence of abusive head trauma [4]. This is associated with mechanical and developmental variances across different age groups, as the skull’s lesser thickness and rigidity in younger ages contributes to increased pliability and reduced protective capacity for the developing brain [5]. Additionally, ongoing neurodevelopment, enhanced brain plasticity, and immaturity of the cerebrovascular autoregulation make young patients vulnerable to developing persistent physical and psychological consequences even following mild TBI (mTBI) [6]. These range from developmental issues and persistent post-traumatic headaches to impairment of cognitive abilities, hindered academic performance and ultimately diminished overall quality of life [7,8,9]. Therefore, prompt diagnosis, accurate severity classification and thorough assessment are of utmost importance in managing these young patients, as they enable timely decision making that can mitigate long-term complications but also reduce costs and optimize recovery outcomes.

Computed tomography (CT) is usually utilized for the diagnosis of pTBI in the emergency department. However, concerns regarding radiation exposure and the associated increased risk of leukemia and other malignancies including brain tumors underscore the urgent need for alternative diagnostic and monitoring tools [10,11]. Magnetic resonance imaging (MRI), although increasingly utilized in emergency settings, has some shortcomings involving time constraints, higher cost, lack of wide availability in the emergency department, and other practical considerations [3,10]. Despite the existence of diagnostic and prognostic models, such as the Glasgow Coma Scale (GCS), the Pediatric Emergency Care Applied to Research Network (PECARN), the Canadian Assessment of Tomography for Childhood Head injury (CATCH), and the Glascow Outcome Score (GOS-Extended) the challenge of identifying clinically significant brain injuries (ciTBI), while minimizing unnecessary CT scans and radiation, persists [10,12,13].

The complex pathophysiology of pTBI, including the injury and degeneration of neuronal and glial cells, damage to neurovascular structures, and subsequent neuroinflammation, particularly in the developing brain, paves the way for the exploration of biomarkers that can refine the detection (especially of mTBI), risk stratification, and management of these cases [1,4,14]. Notably, some biomarkers for TBI in adults have been approved by the FDA and are being incorporated into guidelines put forth by the Scandinavian Neurotrauma Committee [14,15].

Several studies are exploring the utility of neuronal and glial biomarkers such as UCH-L1, GFAP, NfL, NSE, S100B, OPN, tau, and p-tau181 in the serum of pTBI patients [14,15,16]. Higher concentrations of these biomarkers are typically observed in injured vs. non-injured children, with increased levels associated with more severe TBI, and worse outcomes [15]. A recent study provided evidence that concentrations of these biomarkers at specific time points, whether at admission, 24 h, or 48 h post-injury, can be used to distinguish pTBI patients from healthy individuals, stratify mild from other TBI severities, and aid in detecting severe TBI [15]. Moreover, associations between biomarker levels at specific intervals and GOS scores at different follow-up periods (2–6 weeks, 6–9 months, and 12 months), further highlight their potential in prognosing patients with less favorable outcomes both acutely and long-term [15]. Interestingly, the time intervals for sampling vary by biomarker.

In another study, inflammatory and metabolic biomarkers, including neutrophil-to-lymphocyte ratio (NLR), platelet to lymphocyte ratio (PLR), and blood glucose were examined for their ability to distinguish between children with mTBI who had positive imaging findings and those who had no abnormalities on CT scans [17]. Notably, the NLR was associated with positive imaging findings, reflecting the rapid activation and response of neutrophils to head trauma, and their role in triggering the inflammatory cascade. This early immune response may explain the link between elevated NLR and the presence of lesions on CT. In the same study, while the PLR and blood glucose levels did not achieve statistical significance, preliminary associations suggesting a potential relationship between increased PLR, glucose levels, and positive imaging findings appear promising. Unlike previous studies that primarily analyzed blood or serum, Holmes et al. took a novel approach by sampling salivary mRNA [18]. This study focused on the expression of complement system components, including C1QB, C4A, C1QA, and C1S in saliva. Their findings suggest that these molecules are overly expressed in patients suffering from acute post-traumatic headaches (C1QA was increased in patients with persistent post-traumatic headaches), highlighting a potential non-invasive biomarker source for monitoring mTBI-related complications [18].

To date, the clinical applicability of these novel biomarkers in pTBI is impeded by several challenges, including inconsistent funding, the absence of age-standardized biomarker concentrations and established cutoff values. The possible addition of biomarkers to established clinical decision rules may further increase their accuracy. Future research should aim to address these issues by developing robust age-specific or age-independent biomarkers and validating cutoff values. The potential of such biomarkers, particularly in resource-limited environments, offers promising prospects for improving TBI diagnosis and management on a global scale.

## References

[1] Alexiou G.A., Sfakianos G., Prodromou N. (2011). Pediatric head trauma. J. Emerg. Trauma. Shock..

[2] Fullerton J.L., Hay J., Bryant-Craig C., Atkinson J., Smith D.H., Stewart W. (2024). Pediatric traumatic brain injury and Microvascular Blood-Brain barrier pathology. JAMA Netw. Open.

[3] Majdan M., Melichova J., Plancikova D., Sivco P., Maas A.I.R., Feigin V.L., Polinder S., Haagsma J.A. (2022). Burden of traumatic brain injuries in children and adolescents in Europe: Hospital discharges, deaths and years of life lost. Children.

[4] Alexiou G., Kafritsas G., Prodromou N., Alexiou G., Prodromou N. (2022). Abusive Head Trauma. Pediatric Neurosurgery for Clinicians.

[5] Högberg U., Fellman V., Squier W., Wester K., Thiblin I. (2025). Cephalhaematoma in Sweden, a Population-Based Register Study. Acta Paediatr..

[6] Serpa R.O., Ferguson L., Larson C., Bailard J., Cooke S., Greco T., Prins M.L. (2021). Pathophysiology of Pediatric Traumatic Brain Injury. Front. Neurol..

[7] Vanderlind W.M., Demers L.A., Engelson G., Fowler R.C., McCart M. (2022). Back to School: Academic Functioning and Educational Needs among Youth with Acquired Brain Injury. Children.

[8] Whitecross S. (2020). Traumatic Brain Injury in Children: The Psychological Effects of Mild Traumatic Brain Injury. J. Binocul. Vis. Ocul. Motil..

[9] Colagiovanni Morrison A., Hall T.A., Kumar V., Williams C.N. (2023). The Impact of Sleep Disturbances on Health-Related Quality of Life in Children With Acquired Brain Injury After Critical Care. Pediatr. Neurol..

[10] Argyropoulou M.I., Alexiou G.A., Xydis V.G., Adamsbaum C., Chateil J.F., Rossi A., Girard N., Vázquez É., Astrakas L.G. (2020). Pediatric minor head injury imaging practices: Results from an ESPR survey. Neuroradiology.

[11] Rivero R., Curran I.L., Hellmann Z., Carroll M., Hornick M., Solomon D., DiLuna M., Morrell P., Christison-Lagay E. (2025). Unnecessary Scans Lead to Unnecessary Re-scans: Evaluating Clinical Management of Low and Intermediate Risk Pediatric Traumatic Brain Injuries. J. Pediatr. Surg..

[12] Gambacorta A., Moro M., Curatola A., Brancato F., Covino M., Chiaretti A., Gatto A. (2022). PECARN Rule in diagnostic process of pediatric patients with minor head trauma in emergency department. Eur. J. Pediatr..

[13] Atiş G.M., Altay T., Atiş Ş.E. (2022). Comparison of CATCH, PECARN, and CHALICE clinical decision rules in pediatric patients with mild head trauma. Eur. J. Trauma. Emerg. Surg..

[14] Reisner A., Zohdy Y.M., Chern J.J., Blackwell L.S., Lepard J.R., Alawieh A., Verma M.S., Kobeissy F., Mulugeta M.G., Tyndall J.A. (2024). The utility of biomarkers in traumatic brain injuries in children: Opportunities and challenges. J. Neurosurg. Pediatr..

[15] Pareja J.C.M., De Rivero Vaccari J.P., Chavez M.M., Kerrigan M., Pringle C., Guthrie K., Swaby K., Coto J., Kobeissy F., Avery K.L. (2023). Prognostic and diagnostic utility of serum biomarkers in pediatric traumatic brain injury. J. Neurotrauma.

[16] Karamian A., Farzaneh H., Khoshnoodi M., Maleki N., Rohatgi S., Ford J.N., Romero J.M. (2025). Accuracy of GFAP and UCH-L1 in predicting brain abnormalities on CT scans after mild traumatic brain injury: A systematic review and meta-analysis. Eur. J. Trauma Emerg. Surg..

[17] Siempis T., Georgalis P.A., Lianos G., Kafritsas G., Metaxas D., Alexiou E., Zika J., Sotiropoulos A., Alexiou G.A., Voulgaris S. (2023). Blood biomarkers for prediction of positive CT findings in mild traumatic brain injury in paediatric population. J. Integr. Neurosci..

[18] Holmes S.A., Mar’i J., Lemme J., Maallo A.M., Lebel A., Simons L., O’Brien M.J., Zurakowski D., Burnstein R., Borsook D. (2022). Evidence of chronic complement activation in asymptomatic pediatric brain injury patients: A pilot study. Children.

